# *In situ* Raman spectroscopic relative quantitative analysis of sulfur metabolic dynamics in deep-sea microorganisms

**DOI:** 10.1128/spectrum.02059-25

**Published:** 2025-09-25

**Authors:** Wanying He, Ruining Cai, Xiaoxiao Guo, Yitong Zhang, Lianfu Li, Shichuan Xi, Zengfeng Du, Zhendong Luan, Jintao Zhuo, Chaomin Sun, Xin Zhang

**Affiliations:** 1 Laoshan Laboratory 474988, Qingdao, China; 2 CAS Key Laboratory of Experimental Marine Biology & Center of Deep Sea Research, Institute of Oceanology, Chinese Academy of Sciences 605415 , Qingdao, China; 3 University of Chinese Academy of Sciences 74519, Beijing, China; 4 Key Laboratory of Ocean Observation and Forecasting, Key Laboratory of Marine Geology and Environment & Center of Deep Sea Research, Institute of Oceanology, Chinese Academy of Sciences 644808 , Qingdao, China; Ocean University of China, Qingdao, Shandong, China

**Keywords:** quantitative analysis, microorganisms, metabolic process, confocal Raman microscopy, sulfur cycle

## Abstract

Deciphering dynamic regulation of microbial sulfur metabolism in deep-sea environments is critical for understanding global biogeochemical cycles and climate feedback mechanisms. Current analytical approaches face limitations in achieving quantitative, *in situ* visualization of microbial metabolic processes, including susceptibility to environmental interference during sampling and analysis, leading to impaired data accuracy. This study developed an innovative method based on confocal Raman spectroscopy utilizing nitrogen as an internal standard for metabolite quantification. Taking sulfate, which is a major component of seawater and essential for the sulfur cycle, as a model, we quantified it in solid medium and monitored the *in situ* metabolic processes of deep-sea *Erythrobacter flavus* 21-3. The non-invasive technique revealed previously unrecognized light-dependent differences in microbial metabolic patterns between deep-sea and laboratory conditions through spectral visualization and relative quantification. We found that natural light exposure promoted sulfate production and enhanced zero-valent sulfur (cyclooctasulfur S_8_) accumulation near the surface, accompanied by co-enrichment of carotenoids, suggesting the presence of light-driven sulfur metabolic processes. In contrast, dark conditions favored S_8_ storage in the subsurface layers, potentially supported by abundant internal organic carbon sources as energy reserves. These findings may provide new insights into photo-regulated sulfur transformation mechanisms. Our approach establishes an analytical framework for *in situ* quantitative investigation of microbially mediated elemental cycling processes.

**IMPORTANCE:**

Microbial sulfur metabolism in the deep ocean is critical to global biogeochemical cycles, yet its regulatory mechanisms remain poorly understood, largely due to methodological limitations. In this study, we introduce an innovative non-invasive, quantitative approach using confocal Raman spectroscopy with molecular nitrogen (N_2_) as an internal standard, overcoming major obstacles in real-time metabolic monitoring. Our results demonstrate light-dependent adaptations in sulfur metabolism among deep-sea bacteria, unveiling previously unrecognized photo-regulated sulfur transformations that refine our understanding of microbial ecological strategies in these environments. The established analytical framework provides a versatile platform for *in situ* investigation of microbial-driven elemental cycling across diverse extreme ecosystems.

## INTRODUCTION

Sulfur is fundamental to biogeochemical cycling in deep-sea ecosystems (1–4). Within anoxic sediments, where conventional electron acceptors are limited, sulfate serves as a critical microbial electron acceptor, with concentrations reaching 28 mM in seawater (3, 5, 6). Sulfate reduction produces intermediate valence sulfur species, including zero-valent sulfur, which drive interconnected elemental cycles (7, 8). As an important form of zero-valent sulfur, cyclooctasulfur (S_8_) can function as a pivotal metabolic nexus (8–10), operating as both an energy storage vector for microorganisms and a biomarker for active sulfur oxidation (11, 12). Recent Raman spectroscopic detection of S_8_ in South China Sea cold-seep communities, coupled with concentration changes during S_8_ production, confirms the role of microorganisms in sulfide transformation (7, 13). Consequently, quantitative study of sulfate, S_8_, and related sulfur species is essential for deciphering microbial-driven sulfur cycling in deep-sea environments (7, 13, 14).

Extensive research has investigated microbially mediated sulfur cycling processes, predominantly focusing on measuring metabolite kinetics in liquid media using chromatography or chemometrics (7, 15). However, these approaches exhibit significant limitations, including inability to obtain long-term *in situ* data, operational complexity, susceptibility to environmental perturbations during sampling, and compromised accuracy due to sampling heterogeneity and contamination. Solid media provide distinct advantages for investigating microbial processes by enabling direct visualization of colony morphology and spatial distribution of sulfur species (7, 9). Quantitative analysis can be effectively performed by tracking metabolite emergence and calculating associated changes in their area or volume (9, 16). Conversely, this approach presents fundamental challenges for absolute quantification of molecules pre-existing within media, particularly sulfate, which is a ubiquitous seawater constituent. This limitation arises because the quantification method relies on presence-absence criterion rather than concentration gradients, inherently constraining its capacity to quantify originally present constituents. Given that deep-sea microorganisms typically grow attached to solid surfaces, with their physiological activities profoundly influenced by the local microenvironment, solid media‌ provide a more representative simulation of their natural habitat (17). Consequently, developing non-sampling-dependent methods capable of *in situ* quantitative analysis of sulfur dynamics within solid matrices is essential for advancing understanding of microbially mediated sulfur cycling mechanisms.

In recent years, Raman spectroscopy has emerged as a transformative analytical platform for biological detection owing to its unique capacity for *in situ* molecular characterization without sample pretreatment, rapid acquisition times, and capacity for simultaneous multi-component analysis (5, 18–20). Critically, this technique overcomes inherent limitations of conventional methods by enabling long-term *in situ* non-destructive quantification and qualitative analysis within complex matrices (21–23). Its capacity to resolve characteristic vibrational peaks of gaseous, liquid, and solid-phase components facilitates comprehensive multiphase system quantification (13, 16, 24–26). Confocal Raman microscopy further advances these capabilities through superior spatial resolution and detection sensitivity compared to conventional Raman systems (27), permitting visualization of microbial colonization dynamics and metabolite distribution patterns within intact solid medium (16). This approach establishes an essential methodological foundation for quantifying temporal changes in metabolite abundance while enabling quantitative monitoring in solid medium that better represents native microbial habitats.

In our prior research, *Erythrobacter flavus* 21-3 was isolated from deep-sea cold seep sediments (7), and its unique thiosulfate-to-sulfate and S_8_ conversion pathway was characterized with demonstrated (10). To investigate dynamic changes in sulfur-containing components within this strain, confocal Raman spectroscopy was employed for long-term, near-real-time monitoring of *E. flavus* 21-3 growth and sulfur transformations, including quantification of S_8_ production (16). Crucially, whereas S_8_ (metabolic product) was directly quantified, sulfate quantification required innovative normalization due to its persistent presence in solid medium‌. Given sulfate’s role as a key dissolved marine constituent influencing microbial metabolism (5), nitrogen was introduced as an internal standard to enable its quantification in solid-phase systems. To further quantify light-mediated regulation of *E. flavus* 21-3-driven sulfur cycling and evaluate whether conventional laboratory lighting masks intrinsic metabolic processes of deep-sea microorganisms, sulfate quantification coupled with S_8_ visualization analysis was utilized to compare sulfur metabolic profiles under natural light versus dark conditions. The resultant data refine our understanding of light-regulated sulfur transformation processes in *E. flavus* 21-3 and demonstrate the value of this approach for application in microbially mediated elemental cycling processes.

## RESULTS AND DISCUSSION

### Modeling of Raman quantitative analysis

Although the intensity of the Raman signal is positively correlated with the concentration of the measured substance, its intensity is also affected by the intensity of excitation light, the Raman scattering cross-section of the measured substance, and the equipment parameters. According to the Raman spectral intensity normalization theory, the ratio of the Raman characteristic peak areas of substances “a” and “b” in the same measured solution is positively correlated with their corresponding concentrations (28, 29), calculated by the following formula:


(1) $$A_{a}/A_{b}=\left(C_{a}/C_{b}\right)\left(\sigma_{a}/\sigma_{b}\right)\left(\eta_{a}/\eta_{b}\right)=\left(C_{a}/C_{b}\right)\left(F_{a}/F_{b}\right)$$

where *A* represents the area of the Raman characteristic peak of the measured object; *C* represents the concentration of the measured object; *σ* represents the Raman scattering cross-section of the measured object; *η* represents the coefficient value related to the parameters of the measuring equipment; and *F* represents the quantification factor (28).

**Fig 1:**
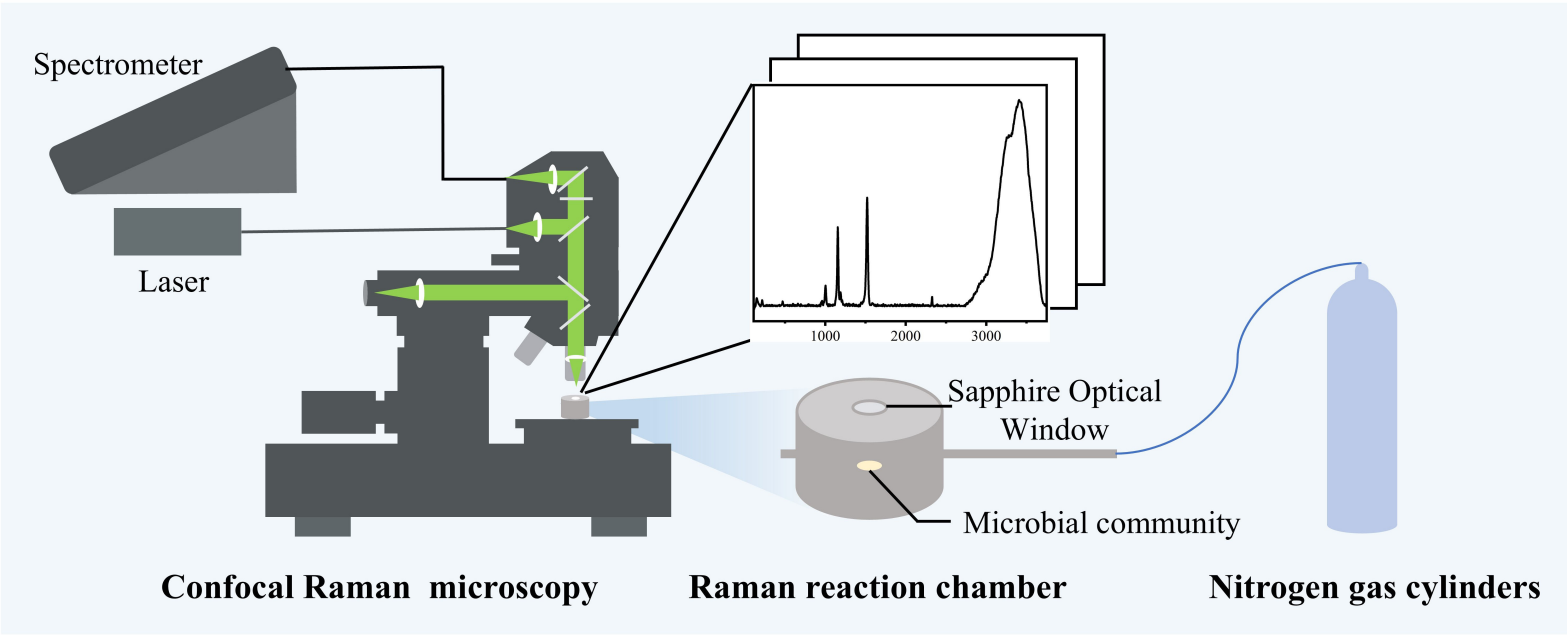
Schematic diagram of Raman quantification of metabolites of colonies in solid medium. The system is equipped with a gas cylinder, a pressure-holding Raman reaction chamber, and a confocal Raman microscopy system.

This calculation method has been widely used for quantitative analysis of liquids or gases (30, 31). For liquid mixtures, water is a commonly used internal standard peak (25, 32, 33), but for the solid medium system in this study where water is involved in microbial metabolism, the water peak was not uniformly stabilized at the later stage. We incidentally found that the stable Raman peak of nitrogen could be measured on the surface of the medium along with the intra-medium components, so we attempted to introduce nitrogen, which was not found to be involved in the reaction and was present stably, as an internal standard. Nitrogen was used to remove all air from the Raman chamber, and the air pressure was maintained at 0.3 MPa to ensure the stability of the nitrogen concentration and to eliminate the possible influence of pressure changes on the detection results (Fig. 1).

In order to demonstrate the feasibility of the methodology, sulfate, which is abundant in seawater and plays an important role in microbially mediated sulfur cycling, was used as a model for the study (5). Much work has been done to quantify sulfate in environments such as conventional conditions and deep-sea extreme environments (13, 33), but little work has been reported to quantify sulfate in solid medium. Since previous studies have found that microbial growth and metabolic processes occur mainly on the surface of the medium, the quantification method in this study only addresses the surface and ignores possible concentration changes within the medium (7, 10). Equal volumes of sterile medium were injected into cultivation vessels while maintaining strictly horizontal alignment to ensure level solidified interfaces following cooling, with optimal focal positioning determined by concurrent maximization of bright-field image clarity and characteristic Raman peak intensities from nitrogen and target analytes. In order to determine the content of sulfate, sodium sulfate solutions (Sinopharm Chemical Reagent Co., Ltd., Shanghai, China) with concentrations of 25 mmol/L–75 mmol/L (purity >99.9%) were collected at the gas-solid interface, respectively, and calibration curves were obtained. The peak area ratio of sulfate molecules to nitrogen molecules was then determined with respect to the concentration of sulfate molecules. For each sample, spectra were collected at the gas-solid interface from at least three distinct positions to minimize experimental errors.

**Fig 2:**
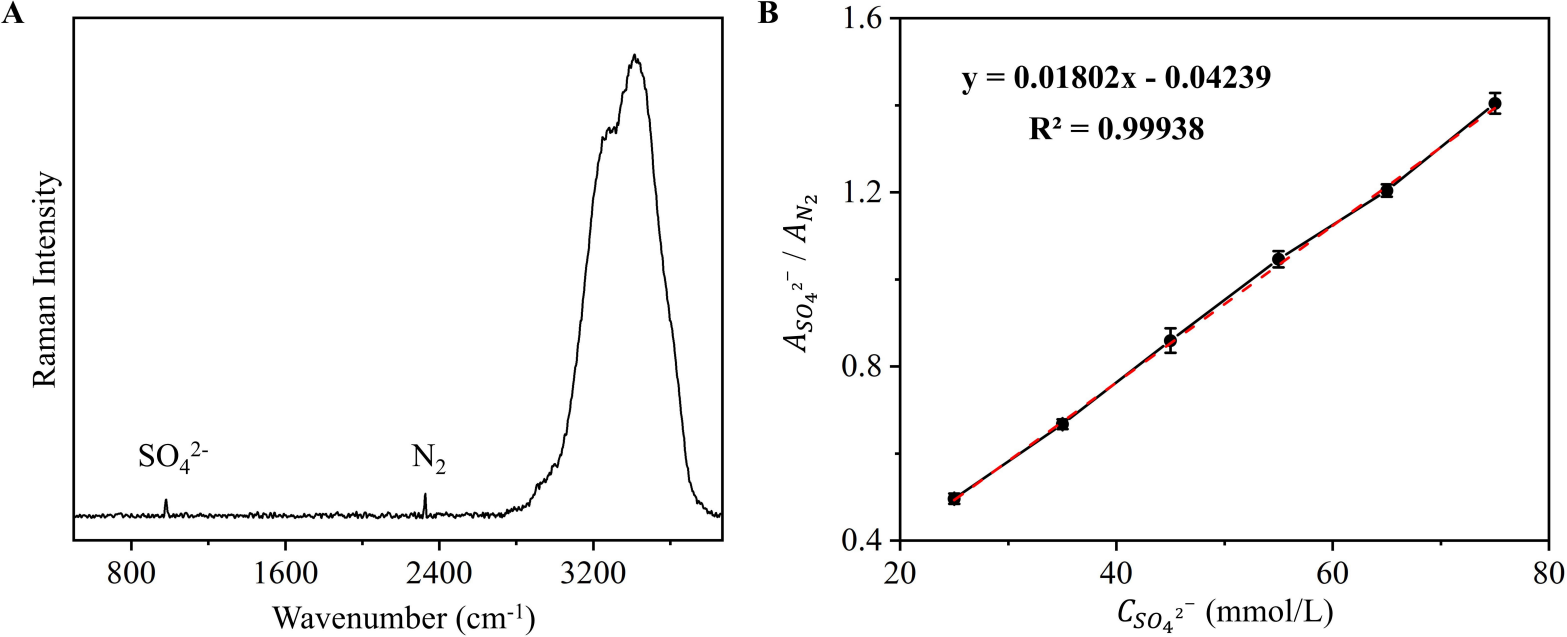
Quantitative modeling of sulfate in solid medium. (**A**) Raman spectra of SO_4_^2−^ and N_2_ at the gas-solid interface of the solid medium. (**B**) Relationship between the concentration of SO_4_^2−^ ($C_{{SO}_{4\;}^{\;2-}}$) and peak area ratio of SO_4_^2−^ and N_2_ ($A_{{SO}_{4\;}^{\;2-}}$/$A_{N_{2}}\;$). Data represent mean ± SD (*n* = 3 replicates). The error bars represent the standard deviation and are visible only when larger than the symbols.


For the fitting analysis, we selected the Raman peaks corresponding to the S-O stretching vibration of sulfate at approximately 980 cm^−^¹ (32) and the N≡N stretching band of nitrogen at 2,332 cm^−^¹ (Fig. 2A) for qualitative and quantitative characterization (34). By Gaussian fitting, we determined the Raman peak areas of sulfate and nitrogen, respectively. The quantitative model is shown in Fig. 2 with the following equations (Table S1):

(2) $$A_{{SO}_{4\;}^{\;2-}}/A_{N_{2}}=0.01802\times C_{{SO}_{4\;}^{\;2-}}-0.04239\left(R^{2}=0.99938\right)$$

where $A_{{SO}_{4\;}^{\;2-}}$/$A_{N_{2}}\;$ denotes the ratio of the peak area of Raman spectra of sulfate and nitrogen molecules to that of the water bending vibration, and $C_{{SO}_{4\;}^{\;2-}}$ denotes the concentration of sulfate in solid medium. This model is mainly adapted to the range of sulfate in solid medium of about 25–75 mmol/L at 0.3 MPa of nitrogen.

### Quantitative Raman analysis of sulfur cycling processes mediated by *E. flavus* 21-3 under natural light conditions

To validate the real-time monitoring capability of sulfate in solid medium, this study systematically characterized dynamic compositional changes during *E. flavus* 21-3-mediated sulfur cycling. The strain’s unique metabolic capacity for thiosulfate disproportionation into sulfate and S_8_, as established in our prior work (7), combined with the critical role of carotenoids in enhancing extremophilic adaptation (35). Specifically, carotenoids maintain membrane integrity, mitigate environmental stressors (e.g., high hydrostatic pressure and low temperature), and provide antioxidant protection under aphotic deep-sea conditions (35). Consequently, beyond tracking sulfate-specific Raman signatures, we detected characteristic peaks of S₈ and carotenoids during later monitoring stages.

**Fig 3:**
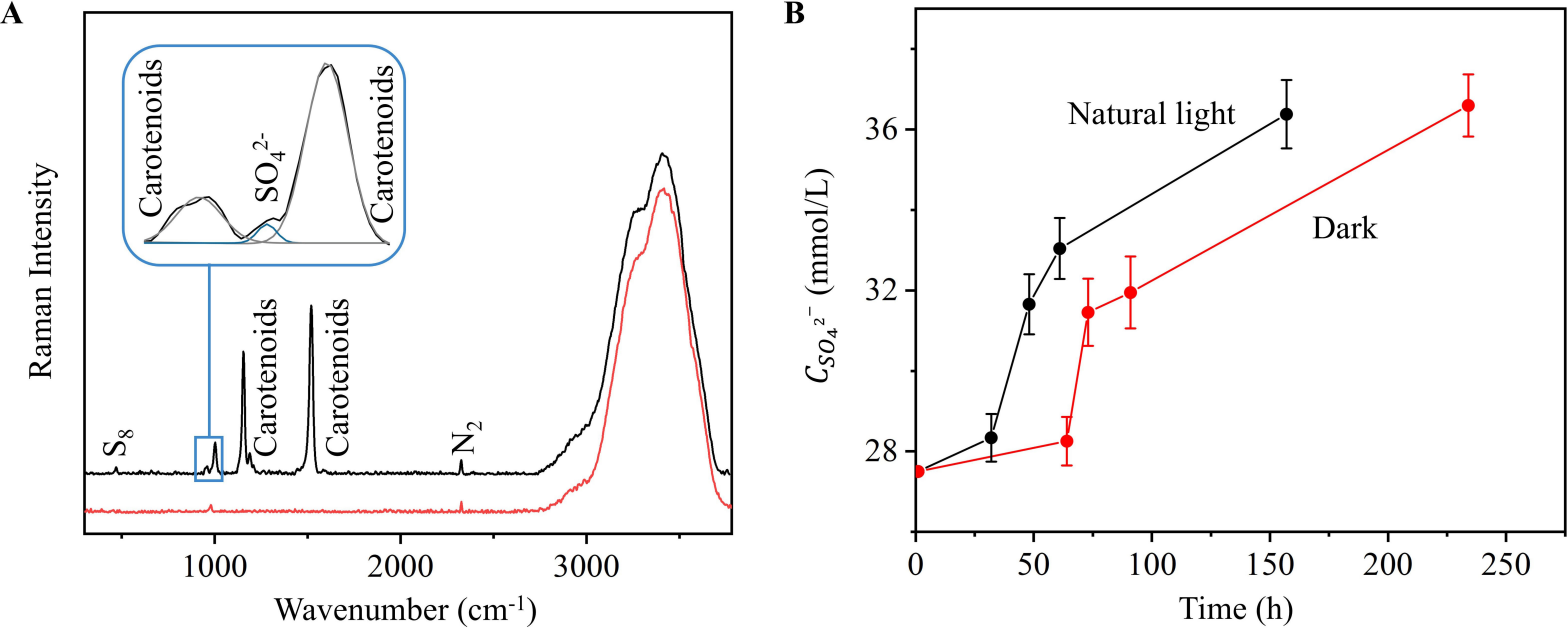
Quantitative analysis of Raman spectra at the gas-solid interface after culturing colonies in solid medium. (**A**) Raman spectra at the interface under natural light (black) and dark (red) conditions. (**B**) Curves of sulfate concentration versus time under natural light (black) and dark (red) conditions. The error bars represent the standard deviation.

**Fig 4:**
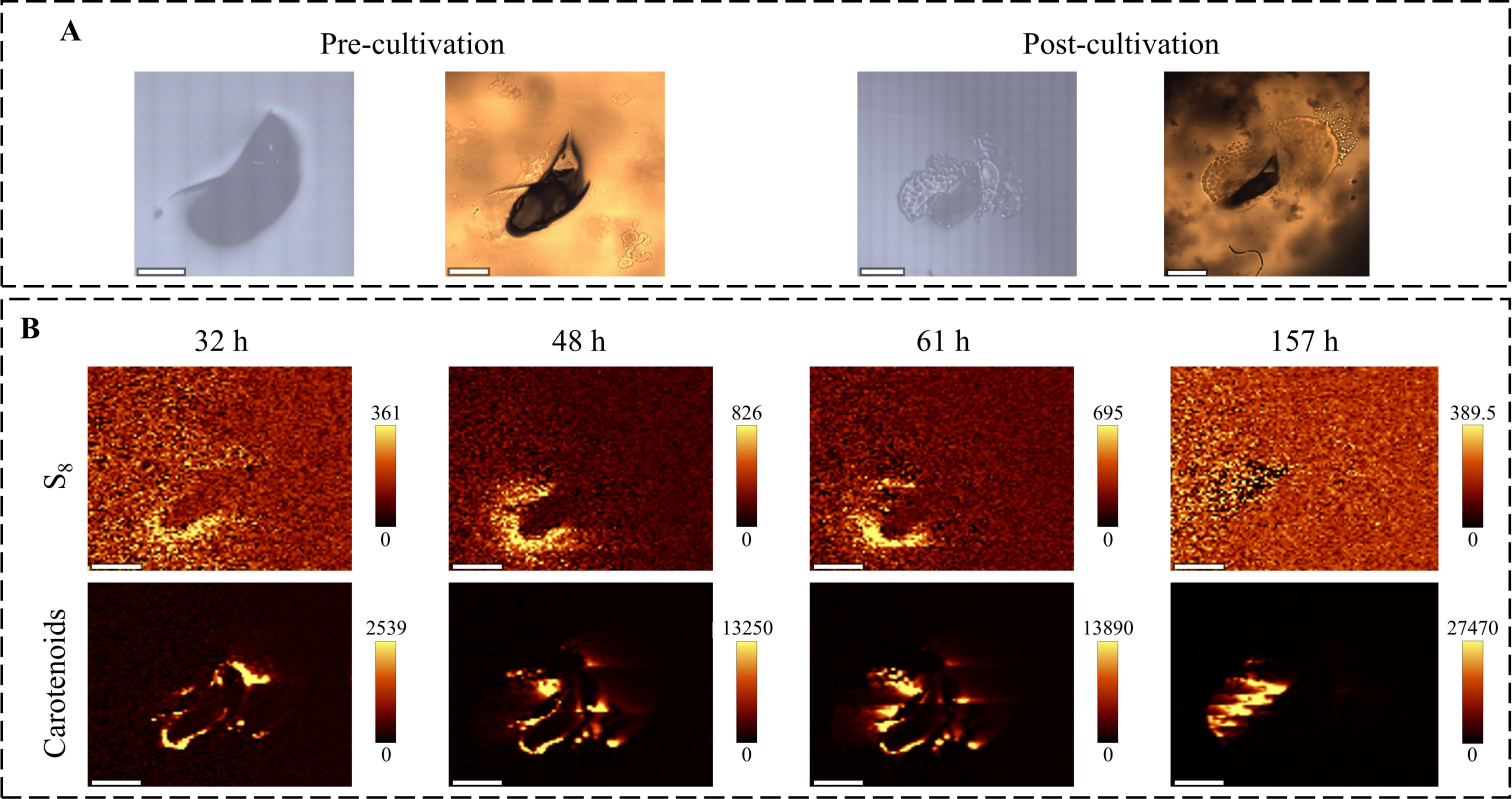
Raman imaging analysis of *E. flavus* 21-3 under natural light. (**A**) Bright-field images of colonies in the early and late stages of culture. Left panel: reflection mode; right panel: transmission mode. Scale bars: 400 μm (pre-cultivation images), 800 μm (post-cultivation images). (**B**) Raman imaging of S_8_ and carotenoids on the surface of the culture medium. S_8_ regions at around 470 ± 10 cm^−1^ and carotenoid regions at around 1,523 ± 10 cm^−1^. Scale bar: 700 µm. The color scale unit of the peak area images is charge-coupled device counts.

Since the Raman peaks of carotenoids were mixed with sulfate, we applied a Gaussian fitting method prior to analysis using the constructed quantitative model (Fig. 3). For S_8_ and carotenoids, which are metabolites that are generated only at a later stage, we performed univariate Raman imaging based on their corresponding vibrational modes to show variations in their content and distribution (Fig. 3 and 4; Fig. S1). The S_8_ was reconstructed using a peak at 470 cm^−1^, which is attributed to the S-S vibration peak (8). And the C = C bond of the polyene chain at 1,523 cm^−1^ was used to represent carotenoids in the medium (Fig. 4; Fig. S1) (14).

During the initial incubation (0–32 h), microbial growth was accompanied by a continuous accumulation of S_8_ and carotenoids (Fig. 4), whereas sulfate production increased moderately (Fig. 3). During the mid-incubation period (32–48 h), S_8_ and carotenoids continued to accumulate, and sulfate production accelerated markedly, suggesting that the increase in biomass facilitated the production of S_8_ and sulfate from thiosulfate. After 48 h, sulfate production declined progressively and was accompanied by a depletion of S_8_, whereas the accumulation of carotenoids continued to slow down. Crucially, after 157 h, carotenoids aggregated within the S_8_ deposition region and continued to accumulate at a near-constant rate (Fig. 4); however, S_8_ consumption decreased significantly. This change in spatial and temporal distribution implies substrate reuse, with S_8_ potentially serving as a substrate for late metabolic maintenance under energy-limited conditions.

Notably, sulfate kinetics were consistent with liquid culture trends, and the S_8_ redistribution pattern matched previous sulfur morphology studies (7, 9, 16). These consistencies validate the reliability of the *in situ* solid-phase quantification method. Overall, this change in spatial distribution and content suggests that the growth of strain *E. flavus* 21-3 is intrinsically linked to sulfur cycling processes.

### Raman quantitative analysis of *E. flavus* 21-3 sulfur metabolic processes under dark conditions

In our previous research, we unexpectedly discovered that light modulates the growth of *E. flavus* 21-3 and its sulfur metabolic pathway (10). Preliminary analysis of the research data indicated that the synthesis of S₈ was enhanced under light conditions, which prompted us to investigate the mechanism underlying this photoresponse. To further quantify the regulatory effect of light on the *E. flavus* 21-3-mediated sulfur cycle and to evaluate whether routine laboratory ambient light masks the intrinsic metabolic processes of this deep-sea microorganism, we established parallel cultivation systems under dark conditions. This comparative approach enabled us to gain further insight into the impact of light regulation on the sulfur metabolic processes in strain 21-3.

**Fig 5:**
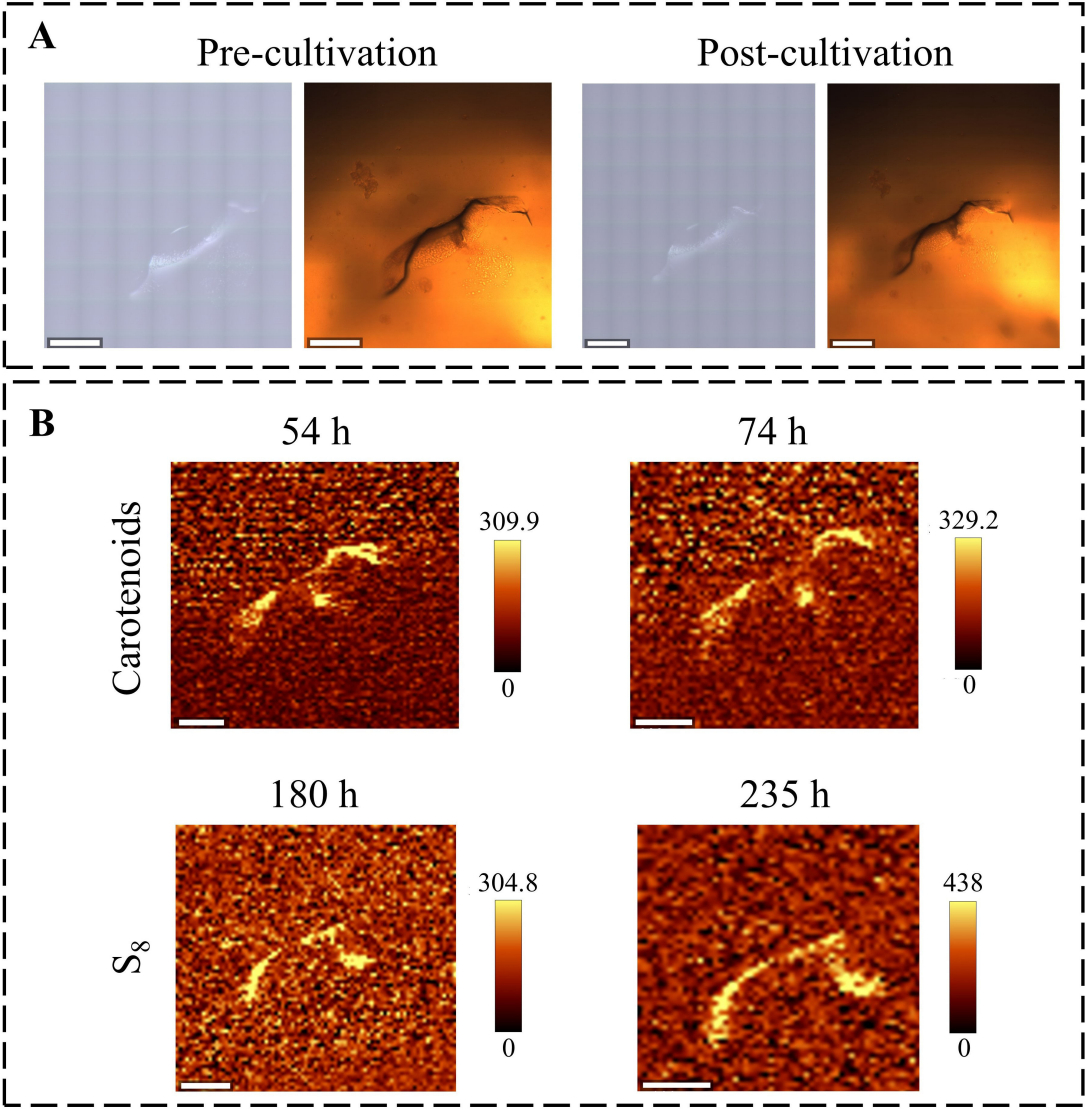
Raman imaging analysis of *E. flavus* 21-3 metabolic processes under dark conditions. (**A**) Bright-field images of colonies in pre-culture/post-culture. Left panel: reflection mode; right panel: transmission mode. Scale bar: 400 µm. (**B**) Raman imaging of S_8_ and carotenoids. S_8_ (470 ± 10 cm^−1^) was localized within subsurface layers, while carotenoids (1,523 ± 10 cm^−1^) accumulated at the culture surface. Scale bar: 300 µm. The color scale unit of the peak area images is charge-coupled device counts.

Under dark conditions, although the trend of the sulfate concentration change curves was similar to that under natural light conditions, with a continuous upward trend, the production rate was significantly slowed down compared with that of colonies cultured under natural light, implying the key role of light in accelerating the metabolic process (Fig. 3). In addition, the synthesis cycle of S_8_ and carotenoids was prolonged, taking more time to reach detectable concentrations. Because the reduced light directly suppressed the production rate of carotenoids (36), the production of carotenoids was detected only at 54 h under dark light conditions (Fig. 5) (37). The absence of detectable carotenoids at later stages may be attributed to the different lifestyles of bacteria that lived in the dark and the light (38, 39).

Notably, while no S₈ production was detected at the medium surface, its formation was observed in the subsurface layer at 180 h (Fig. 5). This spatially specific distribution suggests dynamic metabolic adaptations. Consistent with this, earlier three-dimensional imaging (volumetric mapping across *x*, *y*, *z* axes) data revealed that S_8_ generated by colonies does not persistently deposit on the surface but exhibits longitudinal migration over extended cultivation (16). Under hypoxic conditions, colonies encapsulate sulfur internally (16). These patterns indicate that light, alongside oxygen concentration, serves as a key environmental factor governing sulfur spatial distribution.

In this study, under light conditions, the co-enrichment of carotenoids and S_8_ at the surface likely relies on photo-driven sulfur metabolism. Conversely, lacking photoenergy input, the abundant organic carbon source internal environment favors S_8_ generation as an energy reserve. This light-sulfur metabolism model reveals the unique adaptation mechanism by which deep-sea microorganisms can optimize the utilization of sulfur resources by integrating light-sensing pathways, providing a new perspective for understanding the energy storage strategies of microorganisms in dark ecosystems.

### Conclusion

By establishing an *in situ* quantitative assay coupled with spatiotemporal visualization, this study elucidates microbial growth dynamics and metabolic adaptations under variable light regimes, providing a novel analytical framework for real-time tracking of biogeochemical processes in solid media. Future implementation of integrated three-dimensional Raman scanning with depth-dependent signal correction (40) will significantly enhance the accuracy of spatiotemporal substance distribution profiling and quantitative flux analysis. Coupled with surface-enhanced Raman scattering for low-concentration metabolite detection, this multi-modal platform will be a versatile tool for cross-scale biological studies, advancing the quantification of microbial ecosystem function in the sulfur cycle while enabling mechanistic studies of sulfur sequestration in matrices.

## MATERIALS AND METHODS

### Cultivation of *E. flavus* 21-3

*E. flavus* 21-3 was cultured at 26°C in 2216E medium (7, 9). The medium included 1 g of yeast extract, 5 g of tryptophan, and 15 g of agar in 1 L of artificial seawater, where the artificial seawater contained 24.5 g NaCl, 3.9 g Na_2_SO_4_, 0.7 g KCl, 0.02 g SrCl, 5.0 g MgCl·6H_2_O, 1.1 g CaCl_2_, 0.2 g NaHCO_3_, 0.03 g H_3_BO_4_, and 0.004 g NaF/L per 1 L of Milli-Q water, and the pH was adjusted to a range of 7.2–7.5 with 1 M NaOH. A total of 40 mM sodium thiosulfate was sterilized by 0.22 µm filter membrane and added to autoclaved medium. To ensure experimental reproducibility, standardized initial conditions were rigorously maintained across all trials. After the preparation of the medium, sterile agar was transferred to the Raman reaction chamber linked to a nitrogen bottle. Cultures of *E. flavus* 21-3 were inoculated in the center of the agar medium surface. Continuous nitrogen purge actively displaced atmospheric gases while maintaining precise headspace pressure control at 0.3 MPa. In the experiments with light, the bacteria were exposed to natural light all the time in the laboratory. In the experiments with dark cultivation, the light-transmitting window was covered with a black cloth at all times except for the Raman assay.

### Raman spectra acquisition

A confocal Raman microspectrometer (alpha 300R, WITec, Ulm, Germany) equipped with a laser with an excitation wavelength of 532 nm was used in this study, and an OLYMPUS SLMPlan N 20×/0.25 lens (Olympus Corporation, Tokyo, Japan) was selected for the Raman spectrum acquisition. The equipment utilizes a back-illuminated charge-coupled device camera in conjunction with the UHTS 300 spectrometer. The grating employed is a 600 groove/mm grating, which enables a spectral resolution of 3 cm^−1^. Wavelength calibration is performed by measuring the characteristic Raman peak of a standard single-crystal silicon wafer at 520 cm^−^¹.

For sulfate quantification modeling in solid media, samples with concentration gradients were analyzed in a temperature/pressure-controlled Raman chamber under nitrogen atmosphere at 0.3 MPa. The laser was focused at the gas-solid interface, and spectra were collected from three to five replicate points at different locations to minimize error. The laser power was set to 30 mW to ensure adequate signal intensity while preventing sample phototoxicity. An integration time of 3 s and 60 accumulations was employed to achieve spectra with an appropriate signal-to-noise ratio.

Following the cultivation of inoculated colonies, to ensure data comparability, spectral acquisition was performed based on the colony center. Scanning was conducted within identical spatial ranges relative to the center. The step size, typically set between 20 and 30 µm and proportional to the colony diameter, was chosen to comprehensively capture the spatial distribution of colony components. Concurrently, laser power was reduced to 20 mW and integration time shortened to 1–1.5 s per spectrum to mitigate potential phototoxicity to microbial communities. The subsequent processing protocol involved averaging multiple spatial spectra to compensate for potential degradation in signal-to-noise ratio caused by the shorter integration time and lower power at each point during area scanning, thereby providing high-quality, comparable data for subsequent analysis.

### Processing of Raman spectral data

Data acquired while monitoring colonies were first analyzed using the software WITec Project plus (Control Five 5.2, WITec Company, Ulm, Germany). All Raman data sets were subjected to the same pre-processing, including cosmic ray removal and baseline correction. The details have been meticulously delineated in the study conducted by He et al. (16). Univariate imaging of the vibration patterns of the carotenoid-rich region (1,523 cm^−1^) and cyclooctasulfur S_8_-rich region (470 cm^−1^) was performed using WITec Project plus software. All spectra in the processed data set were then summed and averaged to produce Raman-averaged spectra that are representative of the overall picture. All spectra were finally processed using GRAMS/AI 9.3 (Thermo Fisher Scientific, Inc., Waltham, MA, USA) spectral data processing software. After baseline correction, peak fitting was performed using a Gaussian function to determine band position, width, height, and area for quantitative analysis.

## Data Availability

The data supporting the results of this study are available within the article and in its supplemental information. All other data supporting the findings of this study are available under request to the corresponding author.

## SUPPLEMENTAL MATERIAL

Fig. S1 spectrum.02059-25-s0001.tif Raman quantitative analysis of *E. flavus* 21-3 under natural light.

Table S1 spectrum.02059-25-s0002.xlsx Quantitative modeling of sulfate in solid medium.

ASM does not own the copyrights to Supplemental Material that may be linked to, or accessed through, an article. The authors have granted ASM a non-exclusive, world-wide license to publish the Supplemental Material files. Please contact the corresponding author directly for reuse.
