# Supplementary figures and images for: In situ Raman spectroscopic relative quantitative analysis of sulfur metabolic dynamics in deep-sea microorganisms
Source: Microbiol Spectr. 2025 Sep 25;13(11):e02059-25. doi: 10.1128/spectrum.02059-25 (PMC12584613; doi:10.1128/spectrum.02059-25)

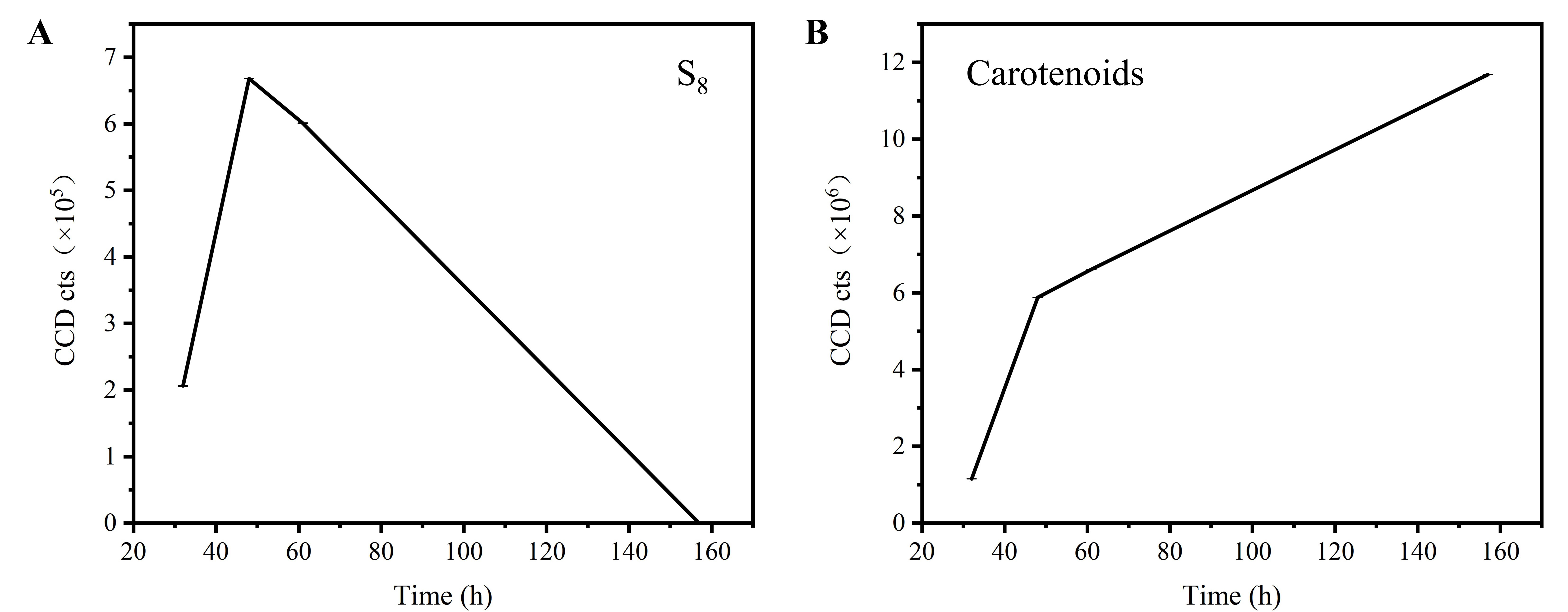

Supplement: Fig. S1 — Raman quantitative analysis of E. flavus 21-3 under natural light. [file spectrum.02059-25-s0001.tif]
